# Epigenetic alterations in type 1 diabetes and their association with poor glycemic control: the SED1-EPI substudy

**DOI:** 10.1038/s41598-026-42995-x

**Published:** 2026-03-13

**Authors:** Ana Victoria García, Carmen Lambert, Elsa Villa-Fernández, Miguel García-Villarino, Isabel Serrano-Olmedo, Elsa Fernández Rubio, Ana Megia Colet, Miguel Brito-Sanfiel, Francisca Payeras Mas, Estefanía Santos, M. Ángeles Martínez de Salinas Santamaría, Fernando Gómez-Peralta, Edelmiro Menéndez-Torre, Pedro Pujante

**Affiliations:** 1 Health Research Institute of the Principality of Asturias (ISPA) Oviedo, Asturias, Spain; 2 Institute of Oncology of the Principaliy of Asturias (IUOPA), University of Oviedo, C/ Julián Clavería s/n. Campus del Cristo, 33006 Oviedo, Asturias Spain; 3 University of Oviedo, Asturias, Spain; 4 Department of Endocrinology and Nutrition, Virgen Macarena Hospital, Sevilla, Spain; 5 Department of Endocrinology and Nutrition, Cruces University Hospital, Barakaldo, Vizcaya Spain; 6 Department of Endocrinology and Nutrition, Research Unit, University Hospital of Tarragona Joan XXIII, Institut d’Investigació Sanitària Pere Virgili (IISPV),, Tarragona, Spain; 7 Department of Endocrinology and Nutrition, Puerta de Hierro University Hospital, 28222 Madrid, Spain; 8 Endocrinology and Nutrition Department, Manacor Hospital, Palma de Mallorca, Spain; 9 Endocrinology and Nutrition Department, University Hospital of Burgos, Burgos, Spain; 10 Endocrinology and Nutrition Department, San Pedro Hospital, Logroño, La Rioja Spain; 11 Endocrinology and Nutrition Department, General Hospital of Segovia, Segovia, Spain; 12 Endocrinology and Nutrition Department, Central University Hospital of Asturias (HUCA). Oviedo, Asturias, Spain; 13 Hospital Univ., Virgen De La Victoria, Spain; 14 Centro de Salud de Barbastro, Hospital Puerto Real, Cádiz, Huesca Spain; 15 Hospital Univ. Virgen De Las Nieves, Granada, Spain; 16 Hospital de Jaén, Jaén, Spain; 17 Hospital Univ. Miguel Servet, Zaragoza, Spain; 18 Hospital Royo Vilanova, Zaragoza, Spain; 19 Hospital Son Llatzer, Mallorca, Spain; 20 Hospital Univ. Nuestra Sra Candelaria, Tenerife, Spain; 21 Hospital General de La Palma, Breña Alta, Spain; 22 Hospital Univ. Marqués De Valdecilla, Santander, Spain; 23 Hospital Clínico Univ. Valladolid, Valladolid, Spain; 24 Complejo Asistencial De Ávila, Ávila, Spain; 25 Complejo Hospitalario Univ. de Albacete, Albacete, Spain; 26 Hospital Univ. de Guadalajara, Guadalajara, Spain; 27 Hospital Nuestra Señora Del Prado, Talavera, Spain; 28 Hospital Sant Pau, Barcelona, Spain; 29 Hospital Univ. Vall Hebrón, Barcelona, Spain; 30 Endocrinology and Nutrition Unit, Hospital Clínic, Barcelona, Spain; 31 Hospital Univ. Bellvitge, Barcelona, Spain; 32 Hospital Germans Trias I Pujol, Barcelona, Spain; 33 Hospital Parc Tauli De Sabadell, Sabadell, Spain; 34 Hospital Arnau De Vilanova De Lleida, Lleida, Spain; 35 Hospital Josep Trueta, Girona, Spain; 36 Hospital de Mataró, Mataró, Spain; 37 Hospital Clinico Univ. De Valencia, Valencia, Spain; 38 Hospital Dr. Peset, Valencia, Spain; 39 Hospital General Univ. Valencia, Valencia, Spain; 40 Hospital General Univ. Alicante, Alicante, Spain; 41 Hospital San Juan De Alicante, Alicante, Spain; 42 Hospital De La Ribera Alzira, Valencia, Spain; 43 Hospital Denia, Denia, Spain; 44 Complejo Hosp. Univ. Castellon, Castellon, Spain; 45 Hospital Gandía, Gandía, Spain; 46 Complejo Hospitalario Univ. Badajoz, Badajoz, Spain; 47 Complejo Hospitalario Univ. Cáceres, Cáceres, Spain; 48 Hospital A Coruña, A Coruña, Spain; 49 Hospital de Ferrol, Ferrol, Spain; 50 Hospital de Vigo, Vigo, Spain; 51 Hospital Clínico de Santiago (Hospital de Conxo), Santiago de Compostela, Spain; 52 Hospital Univ. Gregorio Marañon, Madrid, Spain; 53 Hospital Univ. La Princesa, Madrid, Spain; 54 Hospital Infanta Sofía, San Sebastián de los Reyes, Spain; 55 Hospital Univ. Príncipe de Asturias, Alcalá de Henares, Spain; 56 Hospital Clínico San Carlos, Madrid, Spain; 57 Hospital Infanta Leonor, Madrid, Spain; 58 Hospital Morales Messeguer, Murcia, Spain; 59 Hospital Virgen Arrixaca, Murcia, Spain; 60 Complejo Hospitalario Navarra, Pamplona, Spain; 61 Hospital Univ. Arava, Vitoria, Spain; 62 Hospital Basurto, Basurto, Spain; 63 Hospital Materno-Infantil-H. Regional Univ. de Málaga, Málaga, Spain; 64 Hospital Infantil Virgen del Rocío, Sevilla, Spain; 65 Hospital Univ. Puerta del Mar, Cádiz, Spain; 66 Hospital Miguel Servet, Zaragoza, Spain; 67 Hospital Son Espases, Palma de Mallorca, Spain; 68 Hospital Sant Joan de Deu, Barcelona, Spain; 69 Hospital Vall Hebrón, Barcelona, Spain; 70 Hospital Perpetuo Socorro, Badajoz, Spain; 71 Hospital Univ. Ramón y Cajal, Madrid, Spain; 72 Hospital Univ. La Paz, Madrid, Spain

**Keywords:** Biomarkers, Diseases, Endocrinology, Medical research

## Abstract

Type 1 diabetes (T1D) is an autoimmune disease characterized by β-cell destruction and insulin deficiency. Circulating microRNAs (miRNAs) are promising biomarkers for disease monitoring and complication risk assessment. This study aimed to describe the epigenetic profile in a multicenter Spanish T1D cohort and its association with clinical variables. We analyzed plasma samples from 125 participants (76 T1D, 49 controls) recruited across nine Spanish hospitals. Eight candidate miRNAs from a prior Asturias cohort study were quantified using RT-PCR. Associations with clinical and biochemical parameters were explored, and enrichment analysis was performed to investigate affected biological pathways. Of the miRNAs analyzed, only hsa-miR-200a-3p was significantly overexpressed in T1D versus controls (*p* = 0.035). In T1D patients, hsa-miR-200a-3p, hsa-miR-1-3p, and hsa-miR-340-5p showed positive correlations with HbA1c, while hsa-miR-224-5p correlated with body mass index (BMI). Poor glycemic control (HbA1c ≥ 7.5%) was associated with higher expression of hsa-miR-1-3p, hsa-miR-200a-3p, and hsa-miR-340-5p. No significant expression differences were found according to comorbidity or hypertension status. hsa-miR-200a-3p emerges as a potential biomarker for β-cell stress in long-standing T1D, while hsa-miR-1-3p and hsa-miR-340-5p may reflect glycemic control status and vascular risk. These findings support further evaluation of these miRNAs in larger, clinically diverse T1D cohorts.

**Supplementary Information:**

The online version contains supplementary material available at 10.1038/s41598-026-42995-x.

## Introduction

Type 1 diabetes (T1D) is a chronic autoimmune disorder primarily characterized by the immune-mediated destruction of the insulin-producing beta cells located in the pancreatic islets of Langerhans. This progressive loss of beta-cell function leads to an absolute deficiency of insulin, making individuals with this condition entirely dependent on exogenous insulin therapy to regulate blood glucose levels and maintain metabolic homeostasis^1^. T1D is an increasingly prevalent global health concern, with its incidence rising by approximately 0.34% annually^2^. In 2024, the estimated global incidence was 9.2 million cases^3^.

Despite being a global issue, there are limited studies addressing the incidence and prevalence of T1D in Spain. According to data from the Primary Care Clinical Database (BDCAP), maintained by the Spanish Ministry of Health, the estimated prevalence in 2017 was 0.2%^4^. Regional studies, such as our recent work on the prevalence of T1D in Asturias^5^, and similar efforts conducted in other communities like Madrid^6^, highlight the need to unify data at the national level to obtain a comprehensive picture of the disease across Spain. To address this gap, the Spanish Diabetes Society promoted the SED1 study, whose primary objective was to characterize the clinical profile of Spanish patients with T1D, offer a comprehensive overview of current disease management strategies, and identify factors influencing optimal glycemic control^7^.

Regarding the importance of epigenetics in metabolic-associated diseases, several investigations have focused their attention on the effects of diabetes on the epigenetic profile, mainly regarding type 2 diabetes^8,9^. Regarding T1D, most efforts have been driven to the analysis of their glycemic profile rather than miRNA profile^10,11^.

miRNAs are single-stranded, non-coding RNA molecules (18–24 nucleotides) that play a key role in regulating gene expression post-transcriptionally. Found in all bodily fluids and distributed across every tissue, they are investigated as potential biomarkers, also to predict the prognosis of an already diagnosed disease, or therapeutic targets^12,13^.

Thus, as part of a nested sub-study of the SED1study, plasma samples were collected from a nationally representative cohort to investigate epigenetic differences between individuals with T1D and healthy controls. A previous research by the ENDO-HUCA group examined the differential circulating microRNA (miRNA) expression in people with T1D without comorbidities^14^. In our previous study, mentioned above, we identified in a discovery cohort by next generation sequencing, 22 miRNAs that were differentially expressed between T1D and controls, 5 were downregulated and the other 17 were upregulated. Based on that, we validated the expression of four miRNAs in the whole cohort. Mainly, we could conclude that T1D individuals had higher levels of hsa-miR-1-3p than healthy control individuals. Moreover, a positive correlation between hsa-miR-1-3p expression and HbA1c was observed, suggesting the potential of this miRNA to be an indicator of glycemic control and of cardiovascular risk.

From the 22 miRNAs initially identified, a subset of 8 targets was selected for further analysis based on their high fold-change, statistical power, and potential involvement in diabetic complications. Therefore, the aim of the present study is to validate the expression of these 8 circulating miRNAs in a large, independent multicenter cohort of Spanish adults with long-standing T1D, providing a more robust characterization of their potential as clinical biomarkers.

## Results

### Descriptive

Of the 135 participants in this study, nine were excluded due to shipping issues. The final study cohort consisted of 125 participants, including 76 individuals with T1D and 49 non-diabetic controls. The overall median age was 37 years, with a gender distribution of 53 males and 72 females. Anthropometric and clinical parameters are summarized in Table 1. Individuals with T1D showed significantly higher body mass index (BMI) values compared to controls (24.39 vs. 22.70 kg/m^2^, *p* < 0.05). Median waist circumference and body weight were also slightly higher in the T1D group compared to controls. HbA1c measurements were available exclusively for the T1D group, as these data were not collected for control participants, the median HbA1c in T1D participants was 7.60%, with only 15.78% achieving HbA1c levels ≤ 6.5%.

**Table 1:** Clinical and demographic description and biochemical and glycemic variations.

*N* (Control/T1D) (*N*)	Whole cohort	Control	T1D
	125	49	76
Gender (males/females)	53/72	21/28	32/44
Comorbidities (n)			44/76
Medication information^a^			24/44
HTA			7/68
Age (years)	37.00 [27.75–46.25]	28.00 [27.00–34.00]	45.00 [34.00-52.50]**
Waist (cm)	81.00 [75.5–89.50]	81.00 [76.00-83.75]	88.00 [75.00–96.00]
BMI (kg/m^2^**)**	23.51 [21.69–25.41]	22.70 [21.72–24.51]	24.39 [21.65–26.61]*
Weight (kg)	68.00 [60.00–74.00]	65.00 [59.00–72.00]	69.00 [61.00–76.00]
HbA1c (%)			7.60 [7.10–8.15]
HbA1c (mmol/mol)			60 [54–65]
HbA1c (%) ≤ 6.5 (n [%])			12/76 [15.78]

Data is shown as median [IQ25–IQ75] HbA1c ≤ 6.5% is shown as the percentage of patients with ≤ 6.5 levels.

BMI, Body Mass Index; HTA, Hypertension.

^a^Number of T1D patients with comorbidities, for whom medication use was documented.

p-values correspond to the Mann–Whitney U test for group differences; ***p < 0.001; **p < 0.01; *p < 0.05.

**Table 2:** Analysis of differential miRNA expressions according to the presence or absence of comorbidities and hypertension in T1D.

miRNA	Comorbidities (*p*-value)	HTA (*p*-value)
hsa-miR-141-3p	0.085	0.369
hsa-miR-200b-3p	0.649	0.885
hsa-miR-1-3p	0.474	0.650
hsa-224-5p	0.511	0.600
hsa-340-5p	0.845	0.625
hsa-miR-9-5p	0.070	0.123
hsa-miR-200a-3p	0.533	0.514

p-values for differential miRNA expression according to comorbidities and hypertension status in T1D

### Analysis of plasma circulating levels of different miRNAs

**Fig. 1:**
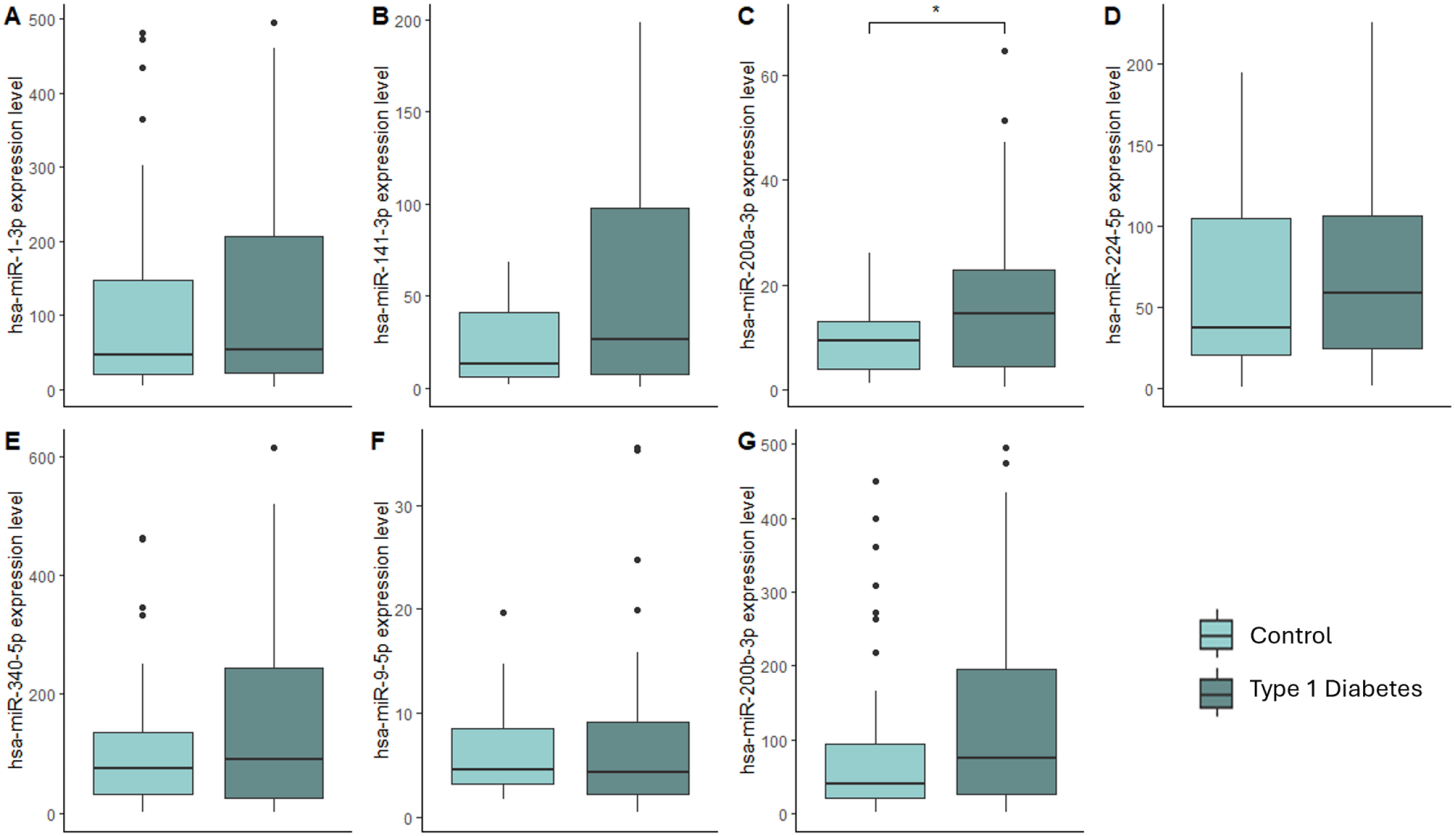
Boxplot images of the seven selected miRNAs (**A**) hsa-miR-1-3p; (**B**) hsa-miR-141-3p; (**C**) hsa-miR-200a-3p; (**D**) hsa-miR-224-5p; (**E**) hsa-miR-340-5p; (**F**) hsa-miR9-5p; (**G**) hsa-miR-200b-3p. Mann–Whitney test was applied for group comparison. *p < 0.05.

We analyzed the expression levels of eight miRNAs comparing the two cohorts (control group vs. people with T1D) (Fig. 1A–G; Supp. Table 3). Among the miRNAs selected for validation, hsa-miR-1299 was excluded, as in the previous study, because more than half of the samples could not be detected by RT-PCR. Although we observed differences in the expression of some miRNAs, hsa-miR-200a-3p was the only one with statistically significant differences between the two groups. Individuals with T1D exhibited higher expression levels of this miRNA compared to the control group (*p* = 0.035; Fig. 1C).

### Plasma circulating levels of miRNAs are correlated with biochemical and anthropometric parameters

**Fig. 2:**
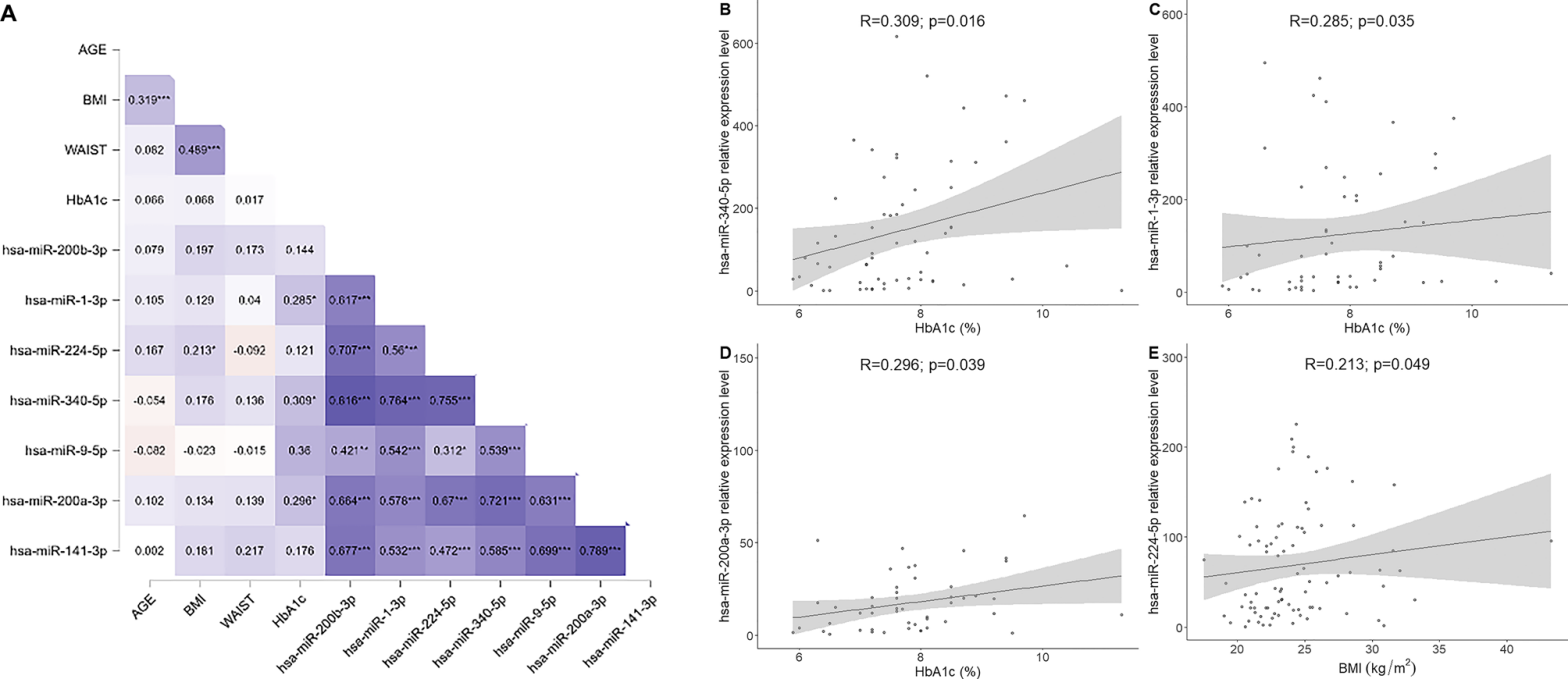
(**A**) Spearman matrix correlation plot between miRNA expression and clinical variables; (**B**) Linear correlation plot between hsa-miR-340-5p and HbA1c percentage; (**C**) Linear correlation plot between hsa-miR-1-3p and HbA1c percentage; (**D**) Linear correlation plot between hsa-miR-200a-3p and HbA1c percentage; (**E**) Linear correlation plot between hsa-miR-224-5p and BMI. BMI body mass index (kg/m^2^), HbAc1 glycated hemoglobin A1c (%) Correlations involving HbA1c were conducted exclusively using data from individuals with T1D.

Correlations between miRNA expression data, biochemical blood parameters, and anthropometric measurements were analyzed (Fig. 2A). A positive correlation was observed between hsa-miR-340-5p, hsa-miR-200a-3p and hsa-miR-1-3p with HbA1c values (Fig. 2B–D). Additionally, a positive correlation was found between hsa-miR-224-5p and BMI (Fig. 2E).

To account for demographic differences between cohorts, a multivariate analysis was performed. After adjusting for age and BMI, hsa-miR-141-3p and hsa-miR-200a-3p showed significant differential expression (*p* < 0.05), suggesting that its levels are influenced by the disease state independently of these clinical variables (Supplementary Table S3).

### Influence of glycemic control in miRNA expression

**Fig. 3:**
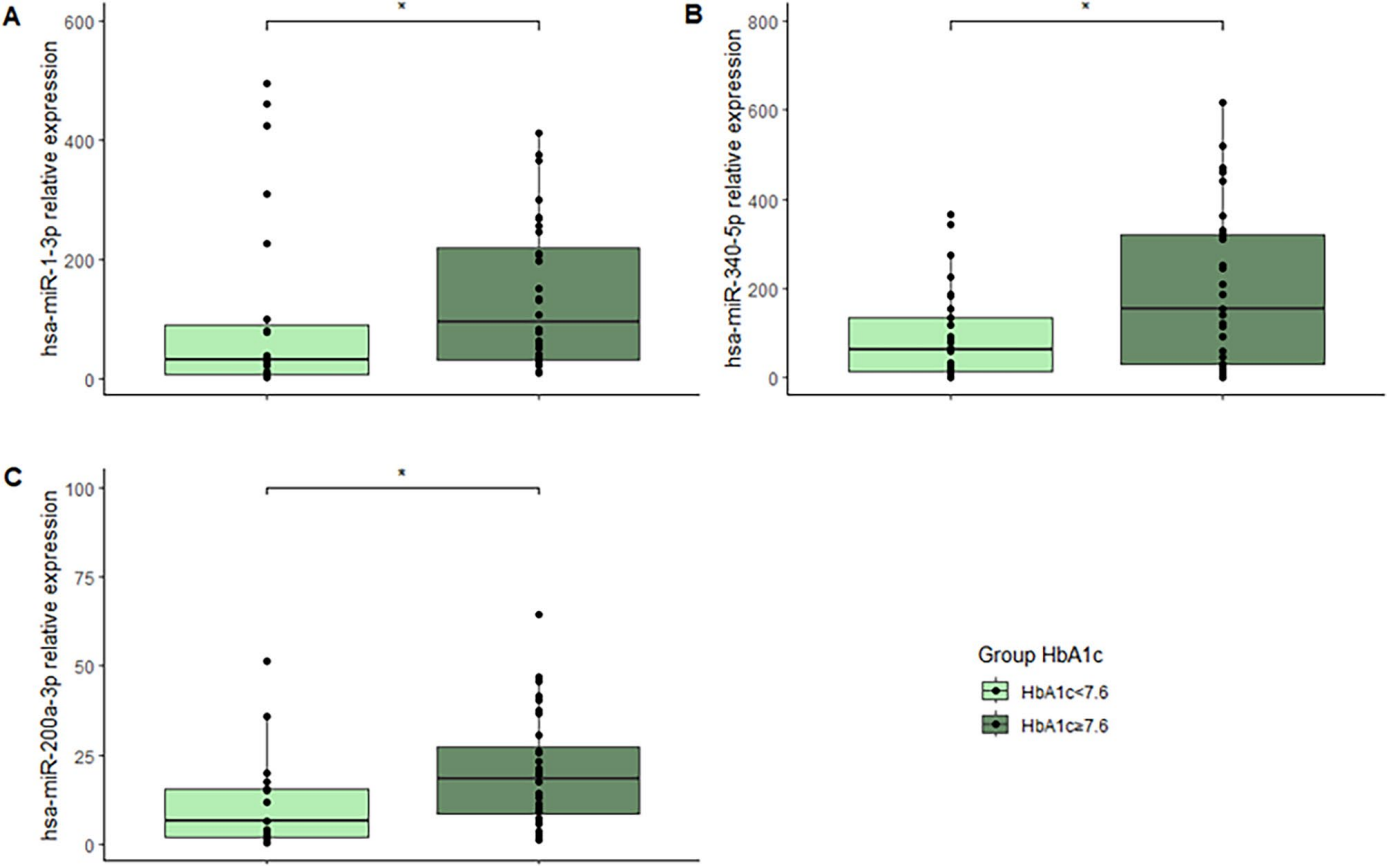
Boxplot images showing miRNA expression in T1D individuals according to their glycaemic control. (**A**) hsa-miR-1-3p; (**B**) hsa-miR-340-5p; (**C**) hsa-miR-200a-3p. *p < 0.050.

Furthermore, all participants in T1D cohort were categorized into groups based on their HbA1c1 levels. A threshold of 7.5% was used to distinguish between glycemic control statuses: individuals with HbA1c1 levels below 7.5% were considered to have better glycemic control, while those with levels equal to or above 7.5% were classified as having worse glycemic control. Individuals with elevated HbA1c levels, exhibited significantly higher expression of hsa-miR-1-3p (*p* = 0.031), hsa-miR-340-5p (*p* = 0.012) and hsa-miR-200a-3p (*p* = 0.026; Fig. 3A-C). By contrast, the expression of the other four miRNAs was not affected by the glycemic status in individuals with T1D.

### Analysis of differential miRNA expression levels according to comorbidity status in T1D individuals

Differences in miRNA expression levels were evaluated among patients with T1D, stratified by the presence or absence of comorbidities and arterial hypertension (HTA). Control subjects were excluded from this analysis as they had no comorbidities.

No statistically significant differences were found in miRNA expression between T1D patients with and without comorbidities and HTA. Although has-miR-9-5p and has-miR-141-3p exhibited a trend toward differential expression according to comorbidity status (*p* < 0.1), these differences did not reach statistical significance.

These findings suggest that, in this cohort of patients with T1D, the presence of comorbidities and HTA is not associated with significant alterations in the expression of the analyzed miRNAs.

### Enrichment

**Fig. 4:**
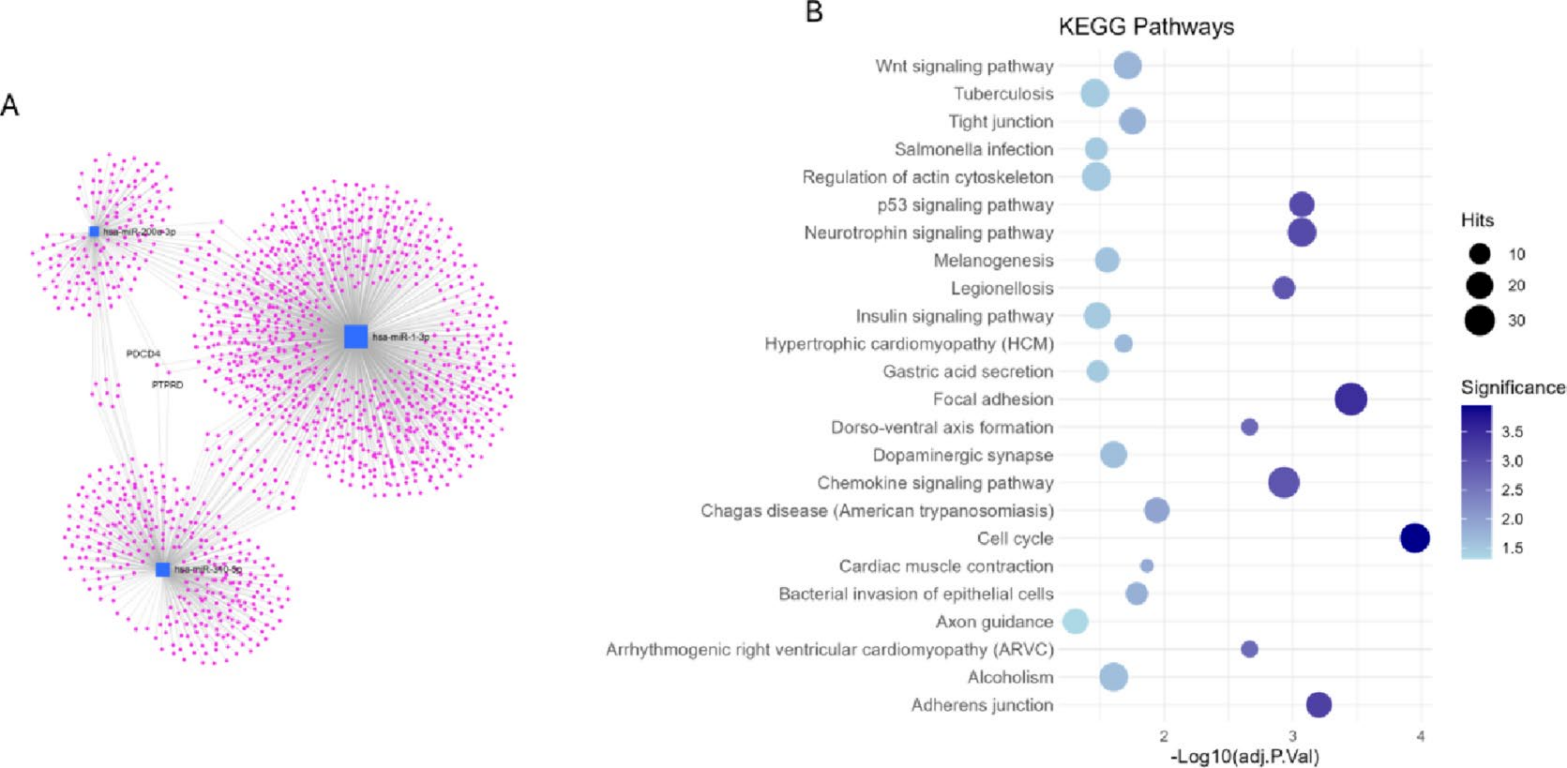
(**A**) Network of the target genes of hsa-mir-1-3p, hsa-mir-340-5p, and hsa-mir-200a-3p. (**B**) Bubble plot of KEGG pathway enrichment analysis for hsa-miR-1-3p, hsa-miR-340-5p and hsa-miR-200a-3p. Bubble size represents the number of associated genes, and color intensity denotes the significance level of the enrichment. Target gene interactions were obtained using the miRNet platform. The complete list of putative target genes is provided in Supplementary Table S4.

Due to the relationship between hsa-miR-200a-3p, hsa-miR-1-3p and hsa-miR-340-5p with glucose metabolism, we performed an enrichment analysis to elucidate the pathways that could be affected by the observed changes. Although only two genes were commonly regulated by these miRNAs (Fig. 4A), many others were shared by at least two of them. This results in the regulation of different pathways, highlighting the insulin signaling pathway and cardiovascular-related pathways, such as the cardiac muscle contraction pathway and the hypertrophic cardiomyopathy pathway (Fig. 4B, 13 and 14).

## Discussion

In this study, we aimed to describe epigenetic circulating miRNA expression patterns in individuals with long-standing T1D by analyzing an independent, multicenter Spanish cohort derived from the SED1 study^7^. Building on our earlier findings in the Asturias cohort, we focused on evaluating the expression of selected miRNAs and exploring their associations with clinical and metabolic parameters relevant to T1D.

Among the eight miRNAs analyzed, only hsa-miR-200a-3p was significantly overexpressed in individuals with T1D compared to healthy controls. Moreover, within the T1D group, hsa-miR-200a-3p levels were positively correlated with HbA1c concentrations, raising the question of whether such epigenetic alterations represent a causal mechanism contributing to poor glycemic control or rather a consequence of sustained hyperglycemia. Previous evidence has shown that epigenetic modifications, particularly DNA methylation, may mediate the development of HbA1c-associated complications in T1D, suggesting a potential role of epigenetic mechanisms in “metabolic memory”^17^ In line with this, our findings are consistent with previous studies implicating this miRNA in β-cell function across different forms of diabetes. Specifically, hsa-miR-200a-3p has been reported to be highly expressed in pancreatic β-cells and associated with their damage and apoptosis. It has been implicated in stress response and anti-apoptotic mechanisms, which contribute to β-cell dysfunction and impaired epithelial-mesenchymal transition. These findings further support the potential role of hsa-miR-200a-3p as a biomarker of β-cell stress and early autoimmune activity in T1D^18^. In agreement with our results, previous studies have also identified hsa-miR-200a-3p as a significantly upregulated marker in the plasma of patients with T1D, suggesting its robust potential as a circulating biomarker for the disease across different cohorts^19^. Our data, showing a positive correlation with HbA1c concentrations, further align with the increasing amount of research indicating the involvement of members of the miR-200 family in insulin secretion, oxidative stress, and inflammation^20^.

In contrast, we were unable to replicate our previous findings regarding hsa-mir-1-3p, which was previously found to be upregulated in individuals with long-standing T1D without complications in the Asturias cohort. In this sub-study, however, 58% of participants presented with diabetes-related complications and were under corresponding pharmacological treatments, potentially influencing the circulating miRNA profile and explaining the discrepancy. Interestingly, while no significant difference in hsa-miR-1-3p expression was observed between T1D and control groups, we did find a significant positive correlation between hsa-miR-1-3p levels and HbA1c in individuals with T1D. This suggests that hsa-miR-1-3p may play a more prominent role in glycemic control than in the presence of T1D per se as we have previously hypothesized.

A similar pattern to that of hsa-miR-1-3p was observed for hsa-miR-340-5p, which, although not differentially expressed between groups, also showed a significant positive correlation with HbA1c levels. This miRNA, although not being widely studied in the field of diabetes, was associated with vascular-related pathologies including diabetic retinopathy^21,22^ and cardiac dysfunction^23,24^. While further studies are warranted to elucidate its role, the strong positive correlations observed for hsa-miR-340-5p, hsa-miR-1-3p, and hsa-miR-200a-3p suggest the possibility of a synergistic effect among them in the regulation of glucose homeostasis and vascular complication. In fact, as we could observe in the enrichment analysis, these miRNAs are strongly related to the insulin signaling pathway and different cardiovascular-related pathways.

As previously mentioned, the main difference between this cohort and the Asturias cohort lies in the presence of comorbidities in the national SED1 cohort. Although these conditions could theoretically impact miRNA expression profiles, all participants in this study were under regular endocrinological follow-up, and their comorbidities were either resolved or controlled under treatment. Therefore, it is not possible to conclusively determine whether the observed differences in miRNA expression are attributable to the presence of comorbidities or to other factors such as treatment regimens or disease progression.

In conclusion, our findings reinforce the potential of hsa-miR-200a-3p as a circulating biomarker for β-cell stress in long-standing T1D and highlight the relevance of hsa-miR-1-3p and hsa-miR-340-5p in glycemic regulation and possibly in the development of diabetic complications. The correlations observed between these miRNAs and HbA1c levels underscore their potential clinical utility in the stratification and monitoring of individuals with T1D. Future studies in larger, well-characterized cohorts, considering treatment status, disease duration, and presence of complications, are needed to further validate these miRNAs as biomarkers and to explore their mechanistic roles in the pathophysiology of T1D and its complications.

This study presents several limitations, most notably regarding the miRNA normalization strategy. While Cel-miR-39 serves as a robust exogenous control for monitoring extraction and reverse transcription efficiency, thereby minimizing pre-analytical variability, it does not account for intrinsic biological input variation. In this substudy, we prioritized analytical consistency; however, the lack of endogenous reference miRNAs, such as hsa-miR-191-5p, remains a constraint. Future validation efforts should incorporate these markers to further refine the accuracy of the expression data.

A limitation of this study is the incomplete availability of detailed clinical, comorbidities and pharmacological information for participants with T1D. As this analysis is based on a registry provided by the Spanish Diabetes Society, access to individualized data on disease duration and specific treatment regimens was restricted. While a substantial proportion of the T1D cohort presented diabetes related comorbidities and were likely under pharmacological treatment, the lack of comprehensive medication data prevented a direct assessment of their impact on circulating miRNA profiles. This limitation may have contributed to the inability to replicate our previous findings on hsa-miR-1-3p observed in a cohort of individuals with long-standing T1D without complications and underscores the need for future studies with more detailed clinical characterization to disentangle the effects of comorbidities and treatment on miRNA expression.

Furthermore, HbA1c levels were only available for the T1D group, as this parameter is not routinely measured in healthy individuals. While this limits the assessment of miRNA associations across the entire study population, the correlations found within the T1D cohort provide valuable insights into the relationship between these miRNAs and glycemic management.

## Materials and methods

### Study population and sample preparation

Volunteers for this study were extracted from the SED1 study, a cross-sectional, multicentre, observational, and minimally invasive investigation, based on retrospective data collected from a nationally representative sample of adults and children with T1D^7^. Specifically, the SED1-EPI study included 135 participants from the original SED1 study: 55 controls and 79 people with T1D, recruited from nine different hospitals across Spain (Supplementary Table 1). The study was conducted in accordance with the principles of the Declaration of Helsinki and received approval from the Spanish Agency of Medicines and Medical Devices (AEMPS), as well as the Research Ethics Committee on Medicinal Products (CEIm) of the Segovia Health Area (Hospital General de Segovia), and from the ethics committees of the other participating hospitals. Written informed consent was obtained from all participants prior to their inclusion in the study. Overnight fating peripheral blood samples were collected in EDTA- containing Vacutainer tubes (BD Biosciences) at the participating hospitals. Blood samples were centrifugated at 2000 rpm for 15 min at 4 °C. Accordingly, all molecular analyses in the present study were centralized at the Research Institute of the Principality of Asturias (ISPA) to ensure methodological consistency and analytical standardization. The top layer containing plasma was divided into aliquots and stored at − 80 °C until their transport to the ISPA.

### miRNAs selection

Based on our previous study conducted in our group^14^, in which we could identify differentially expressed miRNAs in an small cohort of participants from the Principality of Asturias (T1D and controls), we selected eight miRNAs for national validation of the initial findings: hsa-miR-1-3p, hsa-miR-9-5p, hsa-miR-200a-3p, hsa-miR-1299, hsa-miR-200b-3p, hsa-miR-340-5p, hsa-miR-224-5p, hsa-miR-141-3p.

### RNA isolation and DNA transcription

For miRNA analysis, sample preparation was performed as previously described^25^. Briefly, total RNA was extracted from 200 µL of frozen plasma samples using silica membrane columns with the miRNeasy Serum/Plasma Advanced Kit (Qiagen, Hilden, Germany), following the manufacturer’s protocol. A known amount of cel-miR-39 was added during RNA isolation to normalize the results across PCR assays. The purified RNA was eluted in 20 µL of nuclease-free water and stored at − 80 °C until further use.

TaqMan advanced miRNA cDNA synthesis kit (Life Technologies, California, USA) was used to transcribe RNA into cDNA. Gene expression analysis was carried out by RT-PCR using TaqMan^®^ Gene Expression Assays (Applied Biosystems, ThermoFisher, Waltham, MA, USA; Supplementary Table 2) and the Applied Biosystems Prism 7900HT Sequence Detection System (Applied Biosystems) according to the manufacturer’s instructions.

Gene expression data are expressed as target gene RNA expression relative to the corresponding housekeeping mean gene expression (cel-miR-39) (ΔCT = mean CT miRNA - mean CT value of the housekeeping miRNA). The relative expression of each RNA was reported as 2^−ΔCT^.

### Putative gene targets, pathway enrichment analysis and related diseases

Target network, pathway enrichment analysis was performed using miRNet 2.0 web-based tool^26^. miRbase IDs for the three glucose-related miRNAs were uploaded and miRTarBase v9.0 database was selected to annotate experimentally supported target miRNA genes. For functional evaluation, enrichment analysis was conducted using the Kyoto Encyclopedia of Genes and Genomes (KEGG). Cancer-related pathways were excluded, and top affected pathways were represented using RStudio tools.

### Statistical analysis

Descriptive analysis of continuous variables was performed by calculating their median and interquartile range, while categorical variables were expressed as absolute frequencies. Differences in miRNA expression levels between the study groups were assessed using the Mann–Whitney U test, a non-parametric statistical method appropriate for comparing independent samples. To control for potential confounding effects, p-values were adjusted for age, BMI, or both variables using Analysis of Covariance (ANCOVA), as appropriate for each analysis. Correlations were performed using Spearman’s test. A p-value less than 0.05 was considered statistically significant. Statistical analysis was done using JASP version 0.18.1.0 statistical software. All figures were generated in RStudio (2024.12.1 Build 563).

## Supplementary Information

Below is the link to the electronic supplementary material.

Supplementary Material 1

Supplementary Material 2

## Data Availability

The datasets used and/or analysed during the current study are available from the corresponding author on reasonable request.
