# Supplementary material for: Epigenetic alterations in type 1 diabetes and their association with poor glycemic control: the SED1-EPI substudy
Source: Sci Rep. 2026 Mar 13;16:13430. doi: 10.1038/s41598-026-42995-x (PMC13111613; doi:10.1038/s41598-026-42995-x)
Supplement: Supplementary file 1 — Supplementary Material 1 [file 41598_2026_42995_MOESM1_ESM.docx]

| **Suplementary Table 1.** List of hospitals and samples received | |
| --- | --- |
| Hospital | Number of samples |
| Hospital Universitario Virgen Macarena (Sevilla) | 42 |
| Hospital Universitari Joan XXIII de Tarragona | 17 |
| Hospital Universitario de Cruces (Barakaldo) | 18 |
| Hospital Universitario San Pedro (Logroño) | 3 |
| Hospital Puerta del Hierro (Madrid) | 11 |
| Hospital Comarcal de Manacor (Mallorca) | 10 |
| Hospital Universitario de Burgos (Burgos)* | 9 |
| Hospital General de Segovia | 11 |
| Hospital Universitario Central de Asturias (Oviedo) | 14 |
| *Samples received from the Hospital Universitario de Burgos could not be used due to shipping issues. | |

| **Supplementary Table 2.** Taqman miRNA expression assays | | | |  |
| --- | --- | --- | --- | --- |
| **miRNA** | **TaqMan Reference** | **Sequence** | **Observation** |  |
| Cel-miR-39 | 478293_mir | 5´- UCACCGGGUGUAAAUCAGCUUG-3´ | Housekeeping |  |
| hsa-miR-200b-3p | 477963_mir | 5´-UAAUACUGCCUGGUAAUGAUGA-3´ | Selected candidate |  |
| hsa-miR-1-3p | 477820_mir | 5 ́-UGGAAUGUAAAGAAGUAUGUAU-3 ́ | Selected candidate |  |
| hsa-miR-9-5p | 478214_mir | 5´-UCUUUGGUUAUCUAGCUGUAUGA-3´ | Selected candidate |  |
| hsa-miR-1299 | 478696_mir | 5´- UUCUGGAAUUCUGUGUGAGGGA -3´ | Selected candidate |  |
| hsa-miR-200a-3p | 478490_mir | 5´- UAACACUGUCUGGUAACGAUGU-3´ | Selected candidate |  |
| hsa-miR-340-5p | 478042_mir | 5´- UUAUAAAGCAAUGAGACUGAUU-3´ | Selected candidate |  |
| hsa-miR-141-3p | 478501_mir | 5´- UAACACUGUCUGGUAAAGAUGG-3´ | Selected candidate |  |
| hsa-miR-224-5p | 483106_mir | 5´-UCAAGUCACUAGUGGUUCCGUUUAG-3´ | Selected candidate |  |

| **Supplementary Table S3. Statistical comparison of circulating miRNA expression levels between Control and T1D groups.** | | | | |
| --- | --- | --- | --- | --- |
| **miRNA target** | **p-value (unadjusted) ^a^** | **p-value (age-adjusted) ^b^** | **p-value (BMI-adjusted) ^c^** | **p-value (BMI-age-adjusted) ^d^** |
| hsa-200b-3p | 0.189 | 0.253 | 0.469 | 0.452 |
| hsa-miR-1-3p | 0.956 | 0.769 | 0.697 | 0.812 |
| hsa-miR-224-5p | 0.394 | 0.647 | 0.488 | 0.725 |
| hsa-miR-340-5p | 0.409 | 0.232 | 0.389 | 0.375 |
| hsa-miR-9-5p | 0.853 | 0.490 | 0.258 | 0.457 |
| hsa-miR-200a-3p | **0.039** | **0.01** | **0.004** | **0.003** |
| hsa-miR-141-3p | 0.124 | **0.003** | **0.004** | **0.010** |
| **^a^ p-values calculated using the non-parametric Mann-Whitney U test. ^b^ p-values adjusted for Age using Analysis of Covariance (ANCOVA). ^c^ p-values adjusted for Body Mass Index (BMI) using ANCOVA.  ^d^ p-values adjusted for BMI and Age using ANCOVA.  Significant differences (p < 0.05) are highlighted.** | | | | |
